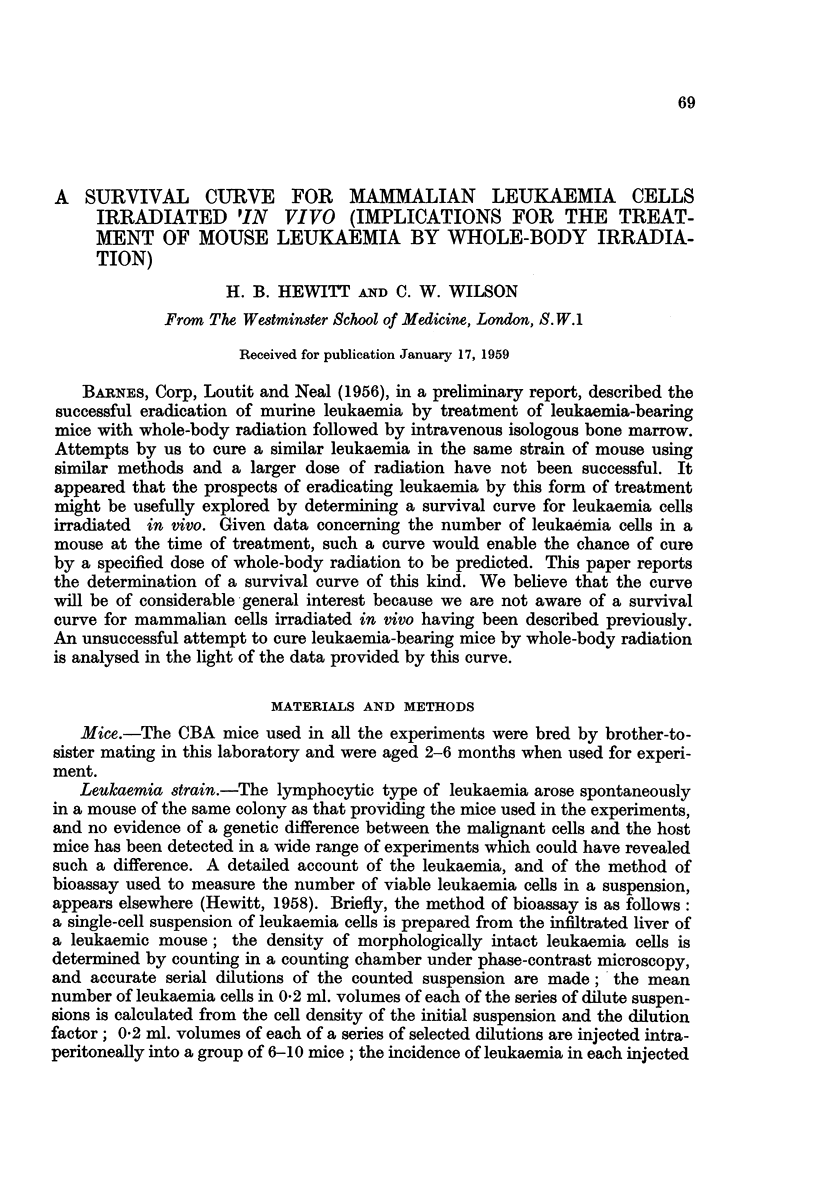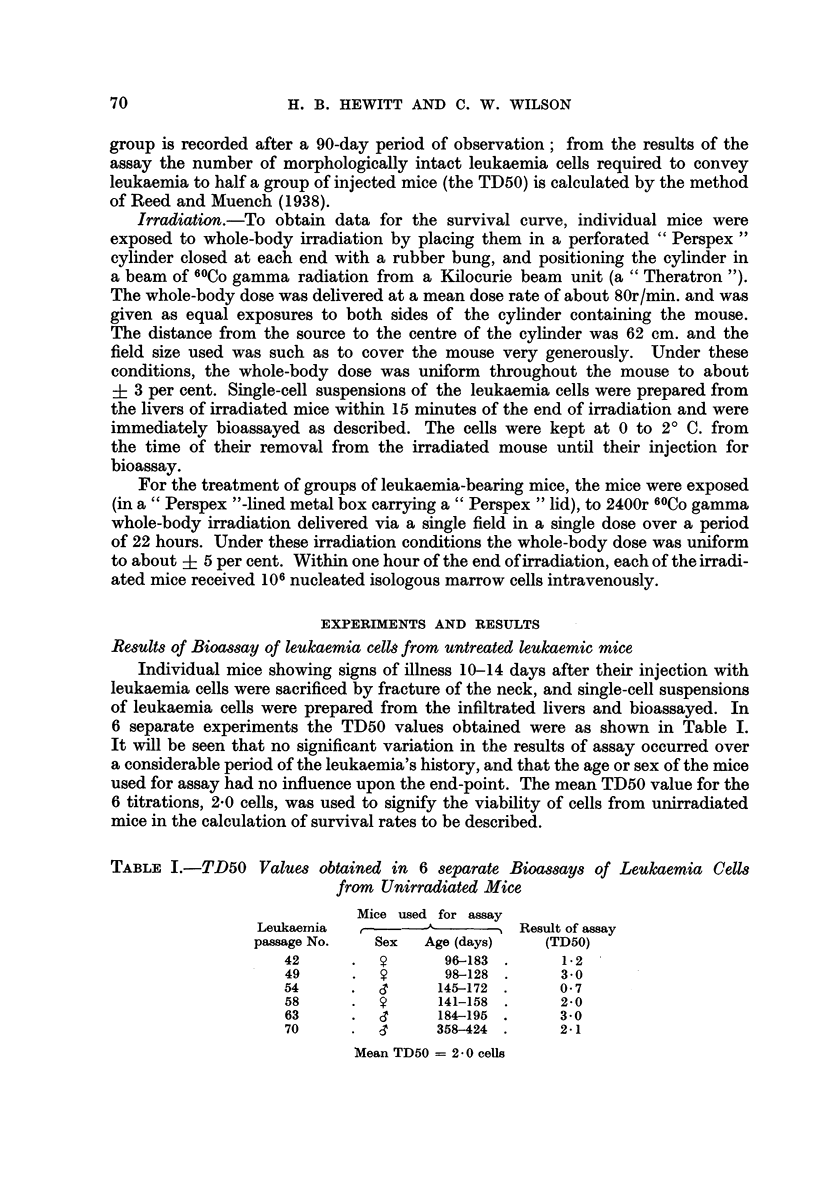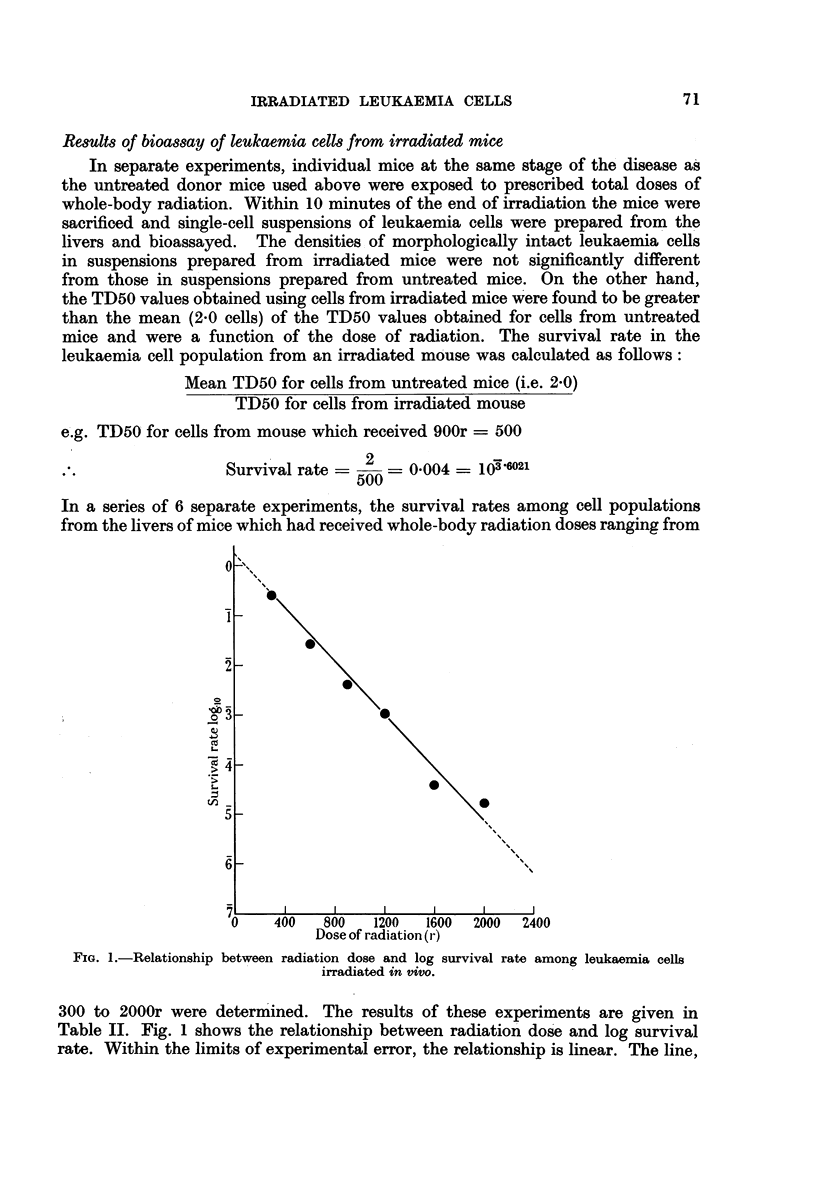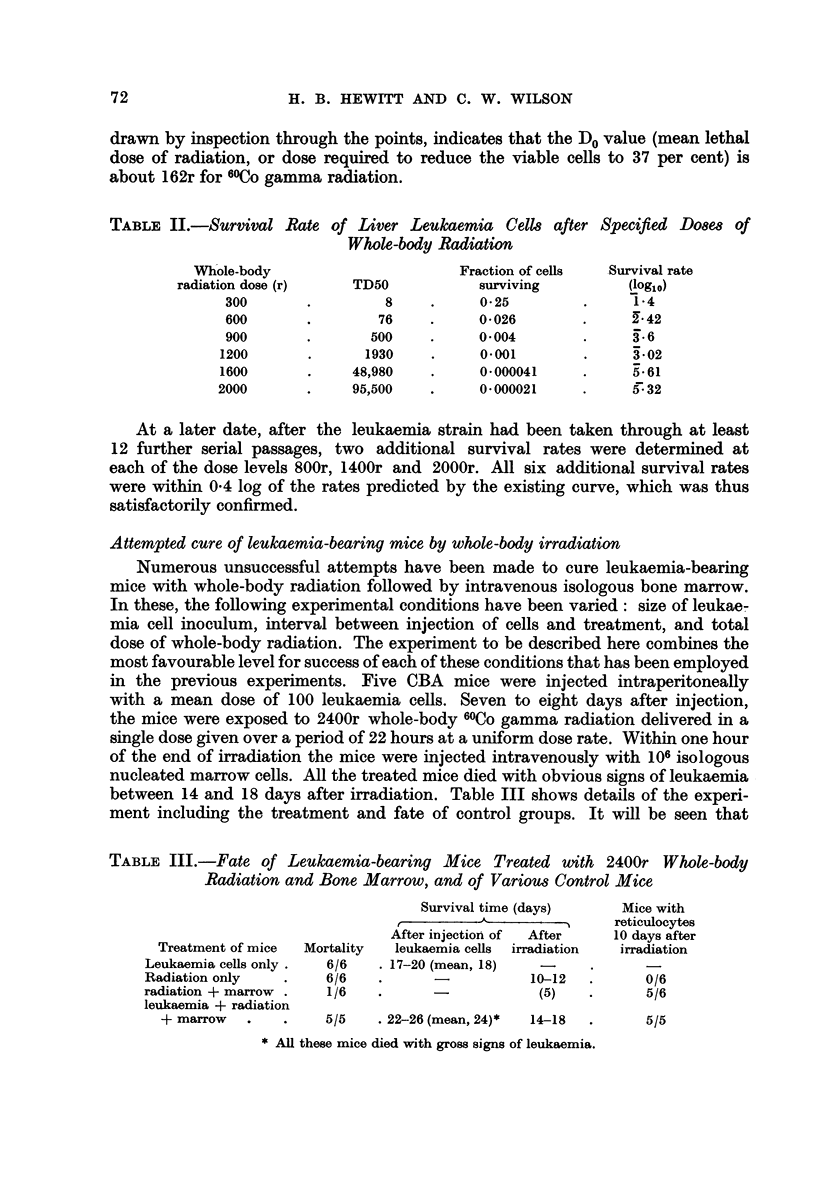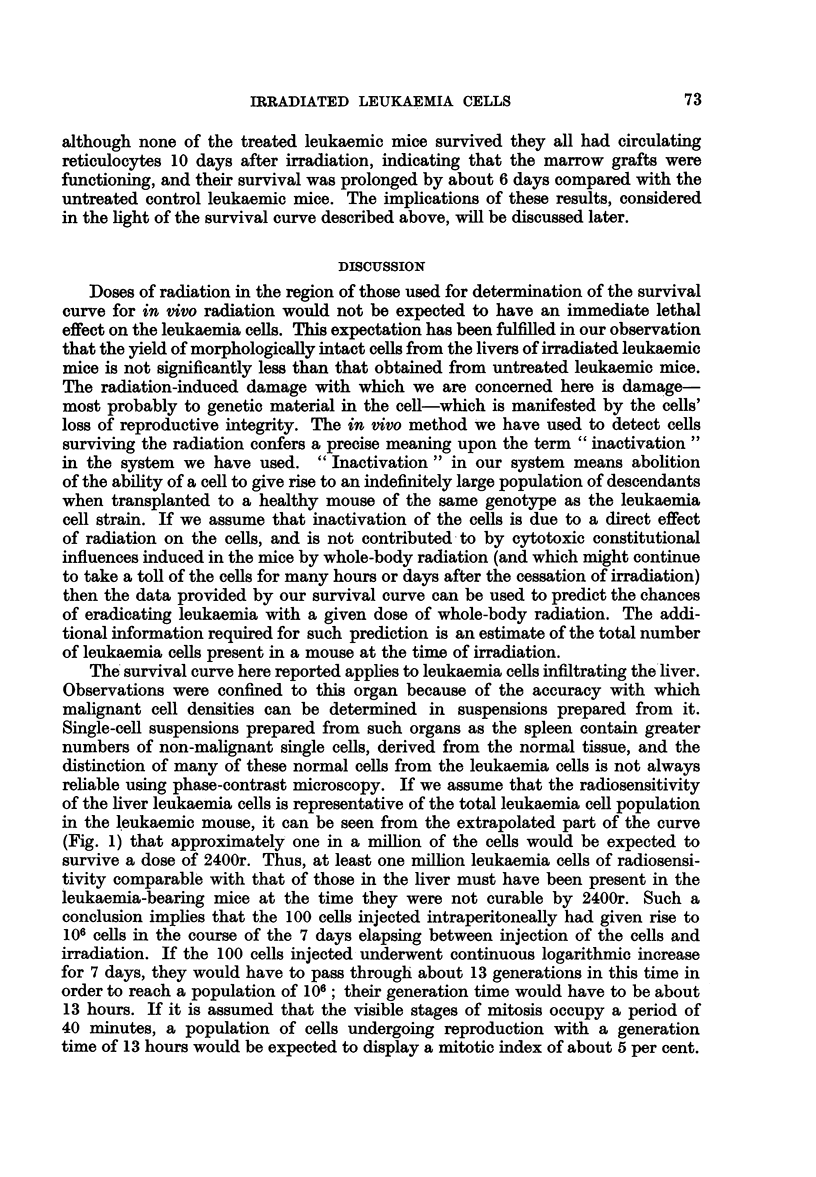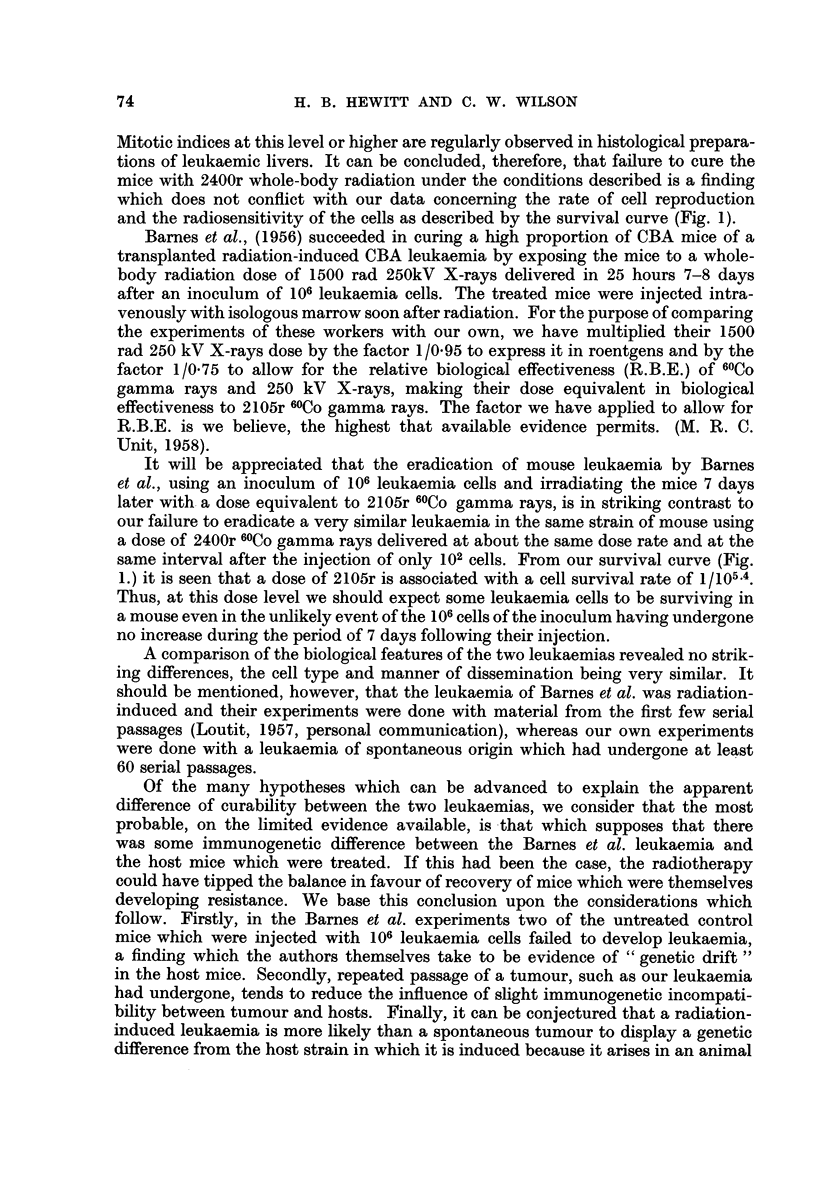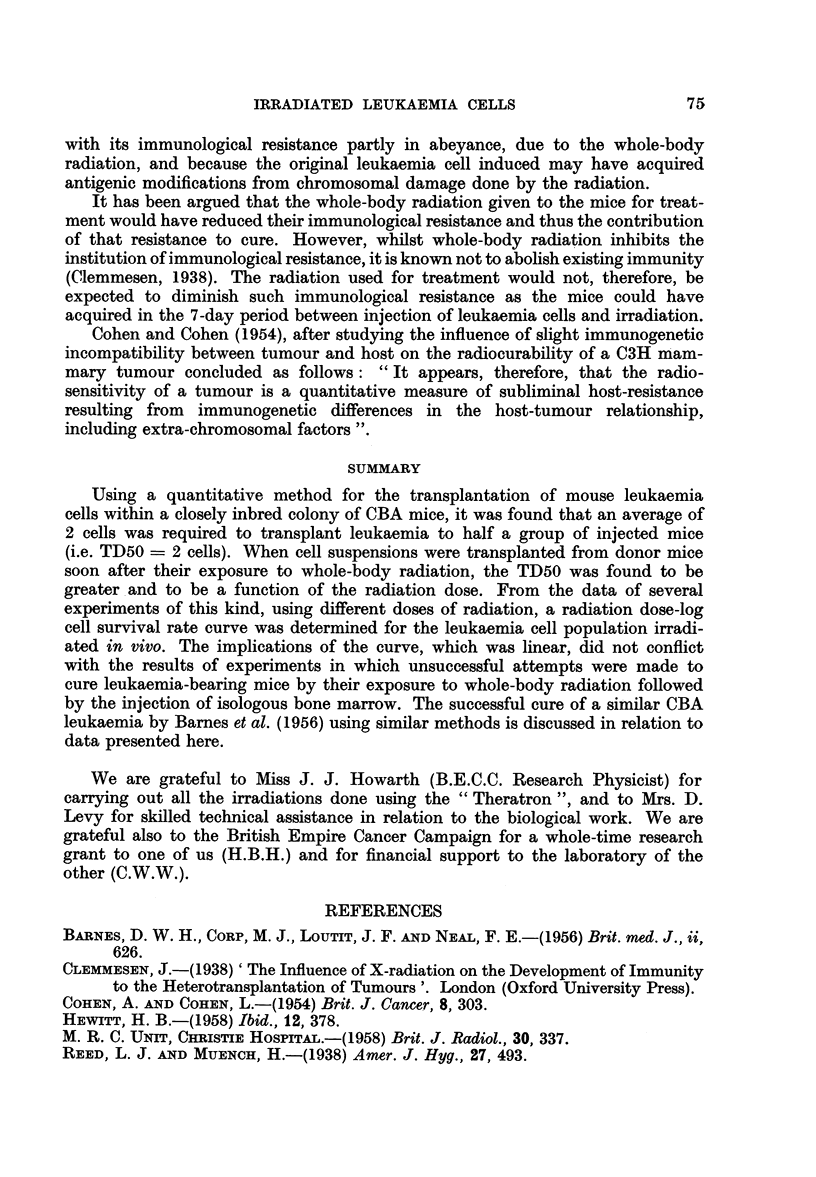# A Survival Curve for Mammalian Leukaemia Cells Irradiated 'in vivo (Implications for the Treatment of Mouse Leukaemia by Whole-Body Irradiation)

**DOI:** 10.1038/bjc.1959.9

**Published:** 1959-03

**Authors:** H. B. Hewitt, C. W. Wilson


### A SURVIVAL CURVE FOR MAMMALIAN LEUKAEMIA CELLS IRRADIATED 'IN VIVO (IMPLICATIONS FOR THE TREATMENT OF MOUSE LEUKAEMIA BY WHOLE-BODY IRRADIATION)

H. B. HEWITT AND C. W. WILSON

*From The Westminster School of Medicine, London, S.W.1*

Received for publication January 17, 1959

Barnes, Corp, Loutit and Neal (1956), in a preliminary report, described the successful eradication of murine leukaemia by treatment of leukaemia-bearing mice with whole-body radiation followed by intravenous isologous bone marrow. Attempts by us to cure a similar leukaemia in the same strain of mouse using similar methods and a larger dose of radiation have not been successful. It appeared that the prospects of eradicating leukaemia by this form of treatment might be usefully explored by determining a survival curve for leukaemia cells irradiated *in vivo*. Given data concerning the number of leukaémia cells in a mouse at the time of treatment, such a curve would enable the chance of cure by a specified dose of whole-body radiation to be predicted. This paper reports the determination of a survival curve of this kind. We believe that the curve will be of considerable general interest because we are not aware of a survival curve for mammalian cells irradiated *in vivo* having been described previously. An unsuccessful attempt to cure leukaemia-bearing mice by whole-body radiation is analysed in the light of the data provided by this curve.

### MATERIALS AND METHODS

*Mice.*—The CBA mice used in all the experiments were bred by brother-to-sister mating in this laboratory and were aged 2–6 months when used for experiment.

*Leukaemia strain.*—The lymphocytic type of leukaemia arose spontaneously in a mouse of the same colony as that providing the mice used in the experiments, and no evidence of a genetic difference between the malignant cells and the host mice has been detected in a wide range of experiments which could have revealed such a difference. A detailed account of the leukaemia, and of the method of bioassay used to measure the number of viable leukaemia cells in a suspension, appears elsewhere (Hewitt, 1958). Briefly, the method of bioassay is as follows : a single-cell suspension of leukaemia cells is prepared from the infiltrated liver of a leukaemic mouse ; the density of morphologically intact leukaemia cells is determined by counting in a counting chamber under phase-contrast microscopy, and accurate serial dilutions of the counted suspension are made ; the mean number of leukaemia cells in 0·2 ml. volumes of each of the series of dilute suspensions is calculated from the cell density of the initial suspension and the dilution factor ; 0·2 ml. volumes of each of a series of selected dilutions are injected intraperitoneally into a group of 6–10 mice ; the incidence of leukaemia in each injected group is recorded after a 90-day period of observation; from the results of the assay the number of morphologically intact leukaemia cells required to convey leukaemia to half a group of injected mice (the TD50) is calculated by the method of Reed and Muench (1938).

*Irradiation.*—To obtain data for the survival curve, individual mice were exposed to whole-body irradiation by placing them in a perforated " Perspex " cylinder closed at each end with a rubber bung, and positioning the cylinder in a beam of $^{60}$Co gamma radiation from a Kilocurie beam unit (a " Theratron "). The whole-body dose was delivered at a mean dose rate of about 80r/min. and was given as equal exposures to both sides of the cylinder containing the mouse. The distance from the source to the centre of the cylinder was 62 cm. and the field size used was such as to cover the mouse very generously. Under these conditions, the whole-body dose was uniform throughout the mouse to about $\pm$ 3 per cent. Single-cell suspensions of the leukaemia cells were prepared from the livers of irradiated mice within 15 minutes of the end of irradiation and were immediately bioassayed as described. The cells were kept at 0 to 2° C. from the time of their removal from the irradiated mouse until their injection for bioassay.

For the treatment of groups of leukaemia-bearing mice, the mice were exposed (in a " Perspex "-lined metal box carrying a " Perspex " lid), to 2400r $^{60}$Co gamma whole-body irradiation delivered via a single field in a single dose over a period of 22 hours. Under these irradiation conditions the whole-body dose was uniform to about $\pm$ 5 per cent. Within one hour of the end of irradiation, each of the irradiated mice received $10^6$ nucleated isologous marrow cells intravenously.

### EXPERIMENTS AND RESULTS

### *Results of Bioassay of leukaemia cells from untreated leukaemic mice*

Individual mice showing signs of illness 10–14 days after their injection with leukaemia cells were sacrificed by fracture of the neck, and single-cell suspensions of leukaemia cells were prepared from the infiltrated livers and bioassayed. In 6 separate experiments the TD50 values obtained were as shown in Table I. It will be seen that no significant variation in the results of assay occurred over a considerable period of the leukaemia's history, and that the age or sex of the mice used for assay had no influence upon the end-point. The mean TD50 value for the 6 titrations, 2·0 cells, was used to signify the viability of cells from unirradiated mice in the calculation of survival rates to be described.

TABLE I.—*TD50 Values obtained in 6 separate Bioassays of Leukaemia Cells from Unirradiated Mice*

| Leukaemia passage No. | Mice used for assay | | Result of assay (TD50) |
|---|---|---|---|
| | Sex | Age (days) | |
| 42 | ♀ | 96–183 | 1·2 |
| 49 | ♀ | 98–128 | 3·0 |
| 54 | ♂ | 145–172 | 0·7 |
| 58 | ♀ | 141–158 | 2·0 |
| 63 | ♂ | 184–195 | 3·0 |
| 70 | ♂ | 358–424 | 2·1 |

Mean TD50 = 2·0 cells

*Results of bioassay of leukaemia cells from irradiated mice*

In separate experiments, individual mice at the same stage of the disease as the untreated donor mice used above were exposed to prescribed total doses of whole-body radiation. Within 10 minutes of the end of irradiation the mice were sacrificed and single-cell suspensions of leukaemia cells were prepared from the livers and bioassayed. The densities of morphologically intact leukaemia cells in suspensions prepared from irradiated mice were not significantly different from those in suspensions prepared from untreated mice. On the other hand, the TD50 values obtained using cells from irradiated mice were found to be greater than the mean (2·0 cells) of the TD50 values obtained for cells from untreated mice and were a function of the dose of radiation. The survival rate in the leukaemia cell population from an irradiated mouse was calculated as follows :

$$\frac{\text{Mean TD50 for cells from untreated mice (i.e. 2·0)}}{\text{TD50 for cells from irradiated mouse}}$$

e.g. TD50 for cells from mouse which received 900r = 500

$$\therefore \qquad \text{Survival rate} = \frac{2}{500} = 0{\cdot}004 = 10^{\bar{3}\cdot 6021}$$

In a series of 6 separate experiments, the survival rates among cell populations from the livers of mice which had received whole-body radiation doses ranging from

FIG. 1.—Relationship between radiation dose and log survival rate among leukaemia cells irradiated *in vivo*.

300 to 2000r were determined. The results of these experiments are given in Table II. Fig. 1 shows the relationship between radiation dose and log survival rate. Within the limits of experimental error, the relationship is linear. The line, drawn by inspection through the points, indicates that the $D_0$ value (mean lethal dose of radiation, or dose required to reduce the viable cells to 37 per cent) is about 162r for $^{60}$Co gamma radiation.

TABLE II.—*Survival Rate of Liver Leukaemia Cells after Specified Doses of Whole-body Radiation*

| Whole-body radiation dose (r) | TD50 | Fraction of cells surviving | Survival rate ($\log_{10}$) |
|---|---|---|---|
| 300 | 8 | 0·25 | $\bar{1}$·4 |
| 600 | 76 | 0·026 | $\bar{2}$·42 |
| 900 | 500 | 0·004 | $\bar{3}$·6 |
| 1200 | 1930 | 0·001 | $\bar{3}$·02 |
| 1600 | 48,980 | 0·000041 | $\bar{5}$·61 |
| 2000 | 95,500 | 0·000021 | $\bar{5}$·32 |

At a later date, after the leukaemia strain had been taken through at least 12 further serial passages, two additional survival rates were determined at each of the dose levels 800r, 1400r and 2000r. All six additional survival rates were within 0·4 log of the rates predicted by the existing curve, which was thus satisfactorily confirmed.

*Attempted cure of leukaemia-bearing mice by whole-body irradiation*

Numerous unsuccessful attempts have been made to cure leukaemia-bearing mice with whole-body radiation followed by intravenous isologous bone marrow. In these, the following experimental conditions have been varied : size of leukaemia cell inoculum, interval between injection of cells and treatment, and total dose of whole-body radiation. The experiment to be described here combines the most favourable level for success of each of these conditions that has been employed in the previous experiments. Five CBA mice were injected intraperitoneally with a mean dose of 100 leukaemia cells. Seven to eight days after injection, the mice were exposed to 2400r whole-body $^{60}$Co gamma radiation delivered in a single dose given over a period of 22 hours at a uniform dose rate. Within one hour of the end of irradiation the mice were injected intravenously with $10^6$ isologous nucleated marrow cells. All the treated mice died with obvious signs of leukaemia between 14 and 18 days after irradiation. Table III shows details of the experiment including the treatment and fate of control groups. It will be seen that

TABLE III.—*Fate of Leukaemia-bearing Mice Treated with* 2400r *Whole-body Radiation and Bone Marrow, and of Various Control Mice*

| Treatment of mice | Mortality | Survival time (days): After injection of leukaemia cells | Survival time (days): After irradiation | Mice with reticulocytes 10 days after irradiation |
|---|---|---|---|---|
| Leukaemia cells only | 6/6 | 17–20 (mean, 18) | — | — |
| Radiation only | 6/6 | — | 10–12 | 0/6 |
| radiation + marrow | 1/6 | — | (5) | 5/6 |
| leukaemia + radiation + marrow | 5/5 | 22–26 (mean, 24)* | 14–18 | 5/5 |

* All these mice died with gross signs of leukaemia.

although none of the treated leukaemic mice survived they all had circulating reticulocytes 10 days after irradiation, indicating that the marrow grafts were functioning, and their survival was prolonged by about 6 days compared with the untreated control leukaemic mice. The implications of these results, considered in the light of the survival curve described above, will be discussed later.

### DISCUSSION

Doses of radiation in the region of those used for determination of the survival curve for *in vivo* radiation would not be expected to have an immediate lethal effect on the leukaemia cells. This expectation has been fulfilled in our observation that the yield of morphologically intact cells from the livers of irradiated leukaemic mice is not significantly less than that obtained from untreated leukaemic mice. The radiation-induced damage with which we are concerned here is damage—most probably to genetic material in the cell—which is manifested by the cells' loss of reproductive integrity. The *in vivo* method we have used to detect cells surviving the radiation confers a precise meaning upon the term " inactivation " in the system we have used. " Inactivation " in our system means abolition of the ability of a cell to give rise to an indefinitely large population of descendants when transplanted to a healthy mouse of the same genotype as the leukaemia cell strain. If we assume that inactivation of the cells is due to a direct effect of radiation on the cells, and is not contributed to by cytotoxic constitutional influences induced in the mice by whole-body radiation (and which might continue to take a toll of the cells for many hours or days after the cessation of irradiation) then the data provided by our survival curve can be used to predict the chances of eradicating leukaemia with a given dose of whole-body radiation. The additional information required for such prediction is an estimate of the total number of leukaemia cells present in a mouse at the time of irradiation.

The survival curve here reported applies to leukaemia cells infiltrating the liver. Observations were confined to this organ because of the accuracy with which malignant cell densities can be determined in suspensions prepared from it. Single-cell suspensions prepared from such organs as the spleen contain greater numbers of non-malignant single cells, derived from the normal tissue, and the distinction of many of these normal cells from the leukaemia cells is not always reliable using phase-contrast microscopy. If we assume that the radiosensitivity of the liver leukaemia cells is representative of the total leukaemia cell population in the leukaemic mouse, it can be seen from the extrapolated part of the curve (Fig. 1) that approximately one in a million of the cells would be expected to survive a dose of 2400r. Thus, at least one million leukaemia cells of radiosensitivity comparable with that of those in the liver must have been present in the leukaemia-bearing mice at the time they were not curable by 2400r. Such a conclusion implies that the 100 cells injected intraperitoneally had given rise to $10^6$ cells in the course of the 7 days elapsing between injection of the cells and irradiation. If the 100 cells injected underwent continuous logarithmic increase for 7 days, they would have to pass through about 13 generations in this time in order to reach a population of $10^6$ ; their generation time would have to be about 13 hours. If it is assumed that the visible stages of mitosis occupy a period of 40 minutes, a population of cells undergoing reproduction with a generation time of 13 hours would be expected to display a mitotic index of about 5 per cent.

Mitotic indices at this level or higher are regularly observed in histological preparations of leukaemic livers. It can be concluded, therefore, that failure to cure the mice with 2400r whole-body radiation under the conditions described is a finding which does not conflict with our data concerning the rate of cell reproduction and the radiosensitivity of the cells as described by the survival curve (Fig. 1).

Barnes *et al.*, (1956) succeeded in curing a high proportion of CBA mice of a transplanted radiation-induced CBA leukaemia by exposing the mice to a whole-body radiation dose of 1500 rad 250kV X-rays delivered in 25 hours 7–8 days after an inoculum of $10^6$ leukaemia cells. The treated mice were injected intravenously with isologous marrow soon after radiation. For the purpose of comparing the experiments of these workers with our own, we have multiplied their 1500 rad 250 kV X-rays dose by the factor $1/0{\cdot}95$ to express it in roentgens and by the factor $1/0{\cdot}75$ to allow for the relative biological effectiveness (R.B.E.) of $^{60}$Co gamma rays and 250 kV X-rays, making their dose equivalent in biological effectiveness to 2105r $^{60}$Co gamma rays. The factor we have applied to allow for R.B.E. is we believe, the highest that available evidence permits. (M. R. C. Unit, 1958).

It will be appreciated that the eradication of mouse leukaemia by Barnes *et al.*, using an inoculum of $10^6$ leukaemia cells and irradiating the mice 7 days later with a dose equivalent to 2105r $^{60}$Co gamma rays, is in striking contrast to our failure to eradicate a very similar leukaemia in the same strain of mouse using a dose of 2400r $^{60}$Co gamma rays delivered at about the same dose rate and at the same interval after the injection of only $10^2$ cells. From our survival curve (Fig. 1.) it is seen that a dose of 2105r is associated with a cell survival rate of $1/10^{5{\cdot}4}$. Thus, at this dose level we should expect some leukaemia cells to be surviving in a mouse even in the unlikely event of the $10^6$ cells of the inoculum having undergone no increase during the period of 7 days following their injection.

A comparison of the biological features of the two leukaemias revealed no striking differences, the cell type and manner of dissemination being very similar. It should be mentioned, however, that the leukaemia of Barnes *et al.* was radiation-induced and their experiments were done with material from the first few serial passages (Loutit, 1957, personal communication), whereas our own experiments were done with a leukaemia of spontaneous origin which had undergone at least 60 serial passages.

Of the many hypotheses which can be advanced to explain the apparent difference of curability between the two leukaemias, we consider that the most probable, on the limited evidence available, is that which supposes that there was some immunogenetic difference between the Barnes *et al.* leukaemia and the host mice which were treated. If this had been the case, the radiotherapy could have tipped the balance in favour of recovery of mice which were themselves developing resistance. We base this conclusion upon the considerations which follow. Firstly, in the Barnes *et al.* experiments two of the untreated control mice which were injected with $10^6$ leukaemia cells failed to develop leukaemia, a finding which the authors themselves take to be evidence of " genetic drift " in the host mice. Secondly, repeated passage of a tumour, such as our leukaemia had undergone, tends to reduce the influence of slight immunogenetic incompatibility between tumour and hosts. Finally, it can be conjectured that a radiation-induced leukaemia is more likely than a spontaneous tumour to display a genetic difference from the host strain in which it is induced because it arises in an animal with its immunological resistance partly in abeyance, due to the whole-body radiation, and because the original leukaemia cell induced may have acquired antigenic modifications from chromosomal damage done by the radiation.

It has been argued that the whole-body radiation given to the mice for treatment would have reduced their immunological resistance and thus the contribution of that resistance to cure. However, whilst whole-body radiation inhibits the institution of immunological resistance, it is known not to abolish existing immunity (Clemmesen, 1938). The radiation used for treatment would not, therefore, be expected to diminish such immunological resistance as the mice could have acquired in the 7-day period between injection of leukaemia cells and irradiation.

Cohen and Cohen (1954), after studying the influence of slight immunogenetic incompatibility between tumour and host on the radiocurability of a C3H mammary tumour concluded as follows: " It appears, therefore, that the radiosensitivity of a tumour is a quantitative measure of subliminal host-resistance resulting from immunogenetic differences in the host-tumour relationship, including extra-chromosomal factors ".

### SUMMARY

Using a quantitative method for the transplantation of mouse leukaemia cells within a closely inbred colony of CBA mice, it was found that an average of 2 cells was required to transplant leukaemia to half a group of injected mice (i.e. TD50 = 2 cells). When cell suspensions were transplanted from donor mice soon after their exposure to whole-body radiation, the TD50 was found to be greater and to be a function of the radiation dose. From the data of several experiments of this kind, using different doses of radiation, a radiation dose-log cell survival rate curve was determined for the leukaemia cell population irradiated *in vivo*. The implications of the curve, which was linear, did not conflict with the results of experiments in which unsuccessful attempts were made to cure leukaemia-bearing mice by their exposure to whole-body radiation followed by the injection of isologous bone marrow. The successful cure of a similar CBA leukaemia by Barnes *et al.* (1956) using similar methods is discussed in relation to data presented here.

We are grateful to Miss J. J. Howarth (B.E.C.C. Research Physicist) for carrying out all the irradiations done using the " Theratron ", and to Mrs. D. Levy for skilled technical assistance in relation to the biological work. We are grateful also to the British Empire Cancer Campaign for a whole-time research grant to one of us (H.B.H.) and for financial support to the laboratory of the other (C.W.W.).

### REFERENCES

BARNES, D. W. H., CORP, M. J., LOUTIT, J. F. AND NEAL, F. E.—(1956) *Brit. med. J.*, *ii*, 626.

CLEMMESEN, J.—(1938) ' The Influence of X-radiation on the Development of Immunity to the Heterotransplantation of Tumours '. London (Oxford University Press).

COHEN, A. AND COHEN, L.—(1954) *Brit. J. Cancer*, **8**, 303.

HEWITT, H. B.—(1958) *Ibid.*, **12**, 378.

M. R. C. UNIT, CHRISTIE HOSPITAL.—(1958) *Brit. J. Radiol.*, **30**, 337.

REED, L. J. AND MUENCH, H.—(1938) *Amer. J. Hyg.*, **27**, 493.